# CD44v8-10 mRNA contained in serum exosomes as a diagnostic marker for docetaxel resistance in prostate cancer patients

**DOI:** 10.1016/j.heliyon.2020.e04138

**Published:** 2020-07-02

**Authors:** Taku Kato, Kosuke Mizutani, Kyojiro Kawakami, Yasunori Fujita, Hidetoshi Ehara, Masafumi Ito

**Affiliations:** a Department of Urology, Gifu University Graduate School of Medicine, Gifu 501-1193, Japan; b Research Team for Mechanism of Aging, Tokyo Metropolitan Institute of Gerontology, Tokyo 173-0015, Japan; c Research Team for Functional Biogerontology, Tokyo Metropolitan Institute of Gerontology, Tokyo 173-0015, Japan; d Department of Urology, Asahi University Hospital, Gifu 500-8523, Japan

**Keywords:** Cell biology, Molecular biology, Cancer research, Chemotherapy, Urology, Castration-resistant prostate cancer, CD44, Docetaxel resistance, Exosomes, Diagnostic marker

## Abstract

**Background:**

Docetaxel is first-line chemotherapy for castration-resistant prostate cancer (CRPC), but most patients acquire docetaxel resistance. CD44 has been shown to be involved in drug resistance of cancers including prostate cancer. We hypothesized that CD44 in serum exosomes could be a diagnostic marker for docetaxel resistance in CRPC patients. In this study, we examined CD44 protein and mRNA expression in cell lysates and exosomes isolated from prostate cancer cells, evaluated the effect of CD44v8-10 knockdown on docetaxel sensitivity and measured CD44 mRNA copy numbers contained in serum exosomes in prostate cancer patients.

**Materials and methods:**

Docetaxel-sensitive PC-3 prostate cancer cells and docetaxel-resistant PC-3R cells established previously from parental PC-3 cells were used. CD44v8-10 knockdown was performed by siRNA transfection. Blood was collected from 50 docetaxel-naïve and 10 docetaxel-resistant patients and 15 control males. CD44 protein expression was evaluated by Western blotting. CD44 mRNA expression was measured by RT-digital PCR.

**Results:**

The levels of CD44v8-10 protein and mRNA in cell lysates and exosomes were higher in PC-3R cells than in PC-3 cells. CD44v8-10 knockdown significantly increased docetaxel sensitivity of PC-3R cells. The CD44v8-10 mRNA copy numbers in serum exosomes were higher in docetaxel-resistant patients than in docetaxel-naïve patients and control males (median 46, 12 and 17 copies/mL serum, respectively, *P* = 0.032). In contrast, the serum exosomal mRNA copy numbers of CD44 standard isoform (CD44s) were not different among 3 groups (median 25, 14 and 13 copies/mL serum, respectively, *P* = 0.150).

**Conclusions:**

CD44v8-10 may be involved in docetaxel resistance in prostate cancer and serum exosomal CD44v8-10 mRNA could be a diagnostic marker for docetaxel-resistant CRPC.

## 1 Introduction

Prostate cancer is the most commonly diagnosed male malignancy and second leading cause of cancer death in the United States [1]. About 5% of prostate cancer patients have distant metastasis at the diagnosis [1]. Androgen deprivation therapy (ADT) is commonly used for metastatic prostate cancer patients. ADT is initially effective in hormone-naïve prostate cancer, but around half of patients progress to castration-resistant prostate cancer (CRPC) following ADT [2, 3]. Several drugs for CRPC have been developed over the past decade [4]. Currently, androgen signaling-targeted therapy (enzalutamide, apalutamide and abiraterone), chemotherapy (docetaxel and cabazitaxel), immunotherapy (sipuleucel-T) and radium-223 are strongly recommended for CRPC treatment according to the National Comprehensive Cancer Network https://www2.tri-kobe.org/nccn/guideline/urological/english/prostate.pdf#search=%27NCCN+prostate%27).

The cluster of differentiation 44 (CD44) is expressed ubiquitously in normal tissues [5] and plays multi-functional roles such as cellular adhesion and hyaluronate degradation [6]. In malignancies, CD44 standard isoform (CD44s) and other alternative splicing variants have been shown to be involved in tumor growth, invasion, migration, metastasis and chemoresistance [7, 8, 9, 10]. Among splicing variant isoforms, CD44 variant 8–10 (CD44v8-10) is reported to play a role in chemoresistance and to be associated with poor prognosis of several types of cancer [11, 12, 13]. In prostate cancer, CD44v8-10 protein expression was increased in cancer tissues than in normal gland tissues and also increased in patients with Gleason grade 3 or higher than in those with grade 1 and 2 [14].

Exosomes are microvesicles with a diameter of 40–150 nm that are released from cells to bodily fluids such as blood and urine. Since exosomes contain DNA, RNA and protein that were present in cells from which they are derived, circulating exosomes are considered as a promising biomarker [15, 16]. In the case of prostate cancer, exosomal androgen receptor splicing variant 7 (ARv7) and P-glycoprotein (P-gp) encoded by *MDR1* gene have been reported to be useful for diagnosis of drug resistance. ARv7 lacks the androgen binding domain and is involved in resistance to androgen-targeted therapy [17, 18]. Del Re *et al.* recently reported that exosomal ARv7 mRNA could be a marker to diagnose resistance to enzalutamide or abiraterone [18]. On the other hand, docetaxel resistance in prostate cancer is partly caused by increased expression of drug efflux pump P-gp [19]. We have previously reported that serum exosomal P-gp has a potential as a diagnostic marker for docetaxel resistance in CRPC patients [20]. Since repeated biopsy is invasive and uncommon in CRPC patients, exosome diagnosis of drug resistance is of particular importance.

In the present study, we hypothesized that CD44 protein and mRNA in serum exosomes could be a diagnostic marker for docetaxel resistance in CRPC patients. First, we examined CD44 protein and mRNA expression in cell lysates and exosomes isolated from docetaxel-resistant and -sensitive prostate cancer cells. Second, we evaluated the effect of CD44v8-10 knockdown on viability of docetaxel-resistant prostate cancer cells. Lastly, we measured CD44s and CD44v8-10 mRNA copy numbers contained in serum exosomes in patients with docetaxel-resistant and -naïve patients and control males.

## 2 Materials and methods

### 2.1 Reagents and antibodies

Docetaxel and paclitaxel were purchased from Sigma-Aldrich (St. Louis, MO). Anti-CD44 and -GAPDH antibodies were obtained from Cell Signaling Technology (Beverly, MA) and anti-CD9 and -MDR1 antibodies were from Santa Cruz Biotechnology (Santa Cruz, CA).

### 2.2 Cell culture

Prostate cancer PC-3 cell line was purchased from the American Type Cell Collection (Manassas, VA). Docetaxel- and paclitaxel-resistant PC-3R cells (PC-3R cells) were generated previously from parental PC-3 cells [20]. PC-3 and PC-3R cells were cultured in RPMI medium with 10% fetal bovine serum in the absence and presence of 20 nM of paclitaxel, respectively.

### 2.3 Isolation of exosomes from cell culture medium

PC-3 and PC-3R cells were cultured in RPMI medium with exosome-free fetal bovine serum for 72 h. Exosomes were isolated from cell culture medium by differential centrifugation as described previously [20].

### 2.4 Western blot analysis

Protein samples were prepared from cells and exosomes as described previously [20]. Ten μg of cell lysates and 5 μg of exosome samples were subjected to electrophoresis on 4–20% sodium dodecyl sulfate-polyacrylamide gels (Bio-Rad, Hercules, CA). Separated proteins were transferred to polyvinylidene difluoride membranes (Millipore, Bedford, MA), which were probed first with primary antibody following blocking in 5% skim milk and then with horseradish peroxidase-linked secondary antibody. Proteins were detected using ImageQuant LAS 4000 mini system (GE Healthcare, Piscataway, NJ). Densitometry was performed in triplicate using Image J software (National Institutes of Health, Bethesda, MD).

### 2.5 Small interfering RNA transfection

Two small interfering RNAs (siRNAs) for CD44v8-10 were designed at Takara Bio (Kusatsu, Japan). Sequences of the siRNAs were as follows: siRNA1 (sense, 5′-CUGACAUCAAGCAAUAGGATT-3′, antisense, 5′-UCCUAUUGCUUGAUGUCAGTT-3′); siRNA2 (sense, 5′-GAAGGUUAUACCUCUCAUUTT-3′, antisense, 5′-AAUGAGAGGUAUAACCUUCTT-3′). As negative control siRNA, Negative Control Medium GC Duplex 2 was used (Invitrogen, Carlsbad, CA). The siRNAs were transfected into cells at 10 nM for 6 h using lipofectamine 2000 (Thermo Fisher Scientific, Waltham, MA).

### 2.6 Cell viability assay

After incubation of cells in 96-well plates for 72 h in the absence or presence of docetaxel, water-soluble tetrazolium salt (WST) assay was performed according to the manufacturer's instruction (Roche, Basel, Switzerland).

### 2.7 Blood collection from patients

Blood was collected from 3 groups of Japanese males: control males in whom prostate cancer was ruled out by prostate biopsy (n = 15), patients with docetaxel-naïve prostate cancer (n = 50) and patients with docetaxel-resistant prostate cancer (n = 10). Patient characteristics were shown in Table 1. Serum was obtained by centrifugation at 1800 × *g* followed by centrifugation at 16,500 × *g* to eliminate cells and stored at -80 °C until use. Docetaxel resistance was diagnosed on the basis of an increasing prostate-specific antigen (PSA) level or radiographic cancer progression during docetaxel administration. This work was approved by the Bioethics Committee of Gifu University (No. 27–146 and 29-6) and written informed consent was obtained from all participants.

**Table 1:** Patient characteristics.

	Control (n = 15)	Docetaxel-naïve (n = 50)	Docetaxel-resistant (n = 10)	*P* value
Age	66 (54–70)	72 (55–81)	75.5 (64–86)	0.002
PSA (ng/ml)	7.014 (3.367–15.436)	8.465 (0.929–16712)	1730.9 (11.128–25313)	<0.001

clinical T				

T1	-	10 (20.0%)	0 (0%)	<0.001
T2	-	24 (48.0%)	3 (30.0%)	
T3	-	9 (18.0%)	5 (50.0%)	
T4	-	6 (12.0%)	1 (10.0%)	
Unknown	-	1 (2.0%)	1 (10.0%)	

Nodal status				

N0	-	42 (84.0%)	5 (50.0%)	0.014
N1	-	6 (12.0%)	5 (50.0%)	
Unknown	-	2 (4.0%)	0 (0%)	

Metastasis				

M0	-	39 (78.0%)	2 (20.0%)	<0.001
M1	-	9 (18.0%)	8 (80.0%)	
Unknown	-	2 (4.0%)	0 (0%)	

Gleason score				

6 ore less	-	12 (24.0%)	0 (0%)	0.003
7	-	19 (38.0%)	0 (0%)	
8 or more	-	17 (34.0%)	8 (80.0%)	
Unknown	-	2 (4.0%)	2 (20.0%)	
Exosomal CD44s (copies/ml)	13 (0–92.3)	14 (0–92.3)	25 (9.7–77.1)	0.150
Exosomal CD44v8-10 (copies/ml)	17 (5.8–1630.7)	12 (0–1630.7)	46 (22.1–145.7)	0.032

### 2.8 RNA extraction and reverse transcriptase-digital PCR

Cells (1 × 10^6^) were cultured in 6-well plates for 48 h. Cellular total RNA was extracted using miRNeasy kit (Qiagen, Valencia, CA) according to the manufacturer's protocol. After filtration through a 0.44-μm membrane (Millipore, Molsheim, France), total RNA in exosomes were directly extracted from serum and cell culture medium using exoRNeasy Serum Plasma kit (Qiagen). Briefly, 4 mL of cell culture medium or 500 μL of serum were applied to the silica membrane included in the kit. Then, attached exosomes were lysed using QIAzol lysis solution. After mixing with chloroform and centrifugation at 12,000 x g for 15 min, total RNA was extracted from the supernatant using RNeasy MinElute spin column. cDNA was synthesized in a total reaction volume of 20 μL using iScript cDNA synthesis kit (Bio-Rad, Hercules, CA). After determination of RNA concentration, 300 ng of cellular total RNA was used in the reaction, whereas the whole amount of exosomal total RNA was subjected to cDNA synthesis in accordance with previously published methods [18, 21, 22]. Digital PCR (dPCR) was performed using 1.5 μL of the cDNA sample according to the manufacturer's instruction (Thermo Fisher Scientific). Briefly, a mixture of cDNA and TaqMan reagents was loaded onto a 20,000-well chip and then PCR amplification was carried out with thermal cycling conditions of 96 °C (10 min), 60 °C (2 min), 98 °C (30 s, 39 cycles) and 60 °C (2 min). Absolute quantification was determined using QuantStudio 3D digital PCR system (Thermo Fisher Scientific).

### 2.9 Statistical analysis

Student *t-*test was used to compare between two parametric groups. For comparing three groups, one-way ANOVA with Fisher's LSD post hoc test or non-parametric Kruskal-Wallis test was used. Chi-square test was used to compare TNM classification and Gleason score between docetaxel-naïve and docetaxel-resistant patients. The data analysis was performed using IBM SPSS Statistics version 23 software (Armonk, NY). *P* < 0.05 was considered statistically significant.

## 3 Results

### 3.1 Increased CD44v8-10 protein expression in cell lysates and exosomes isolated from PC-3R cells

**Figure 1:**
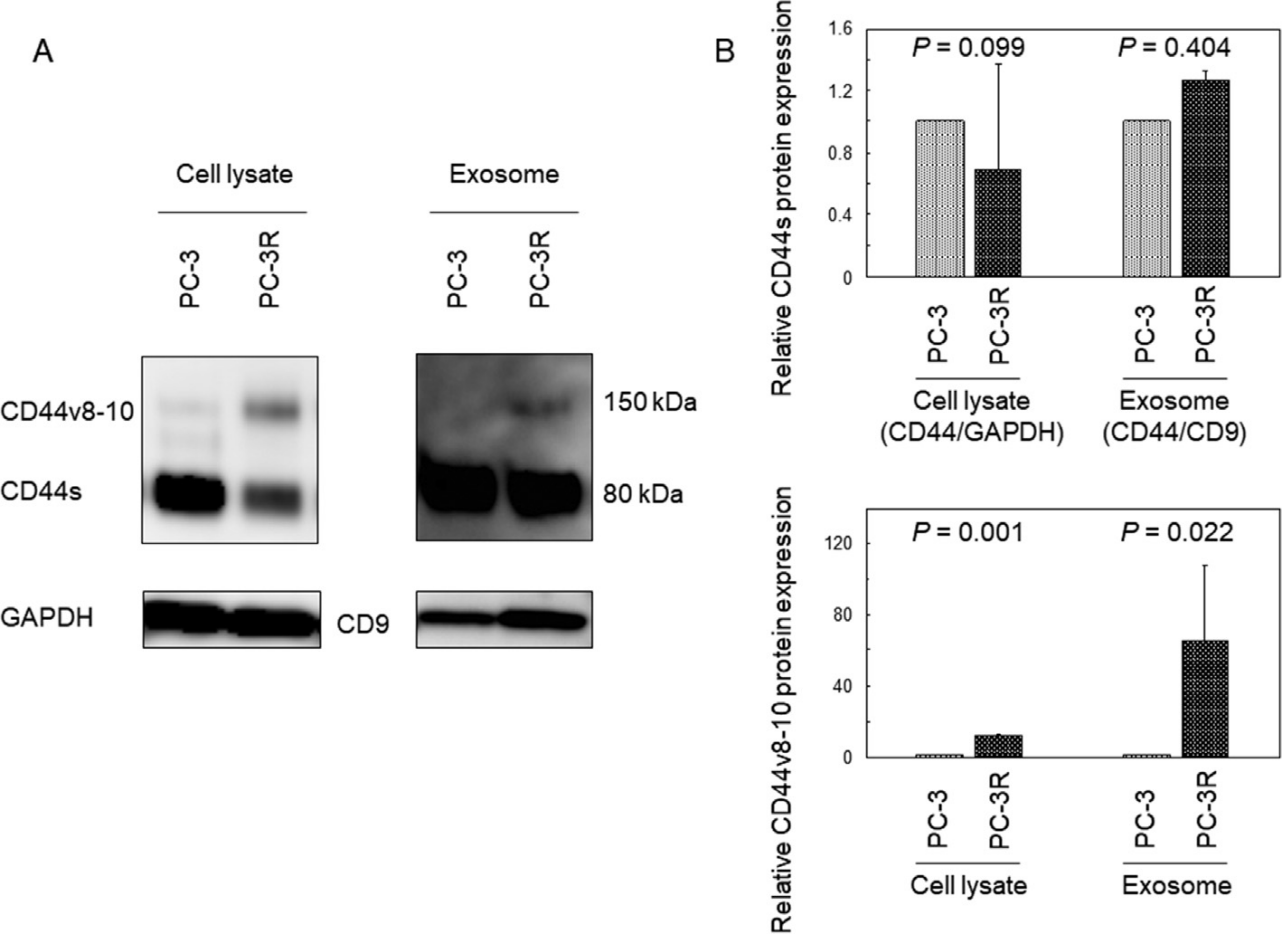
Expression of CD44 proteins in cell lysates and exosomes isolated from PC-3R and parental PC-3 cells. (A) Cell lysates were subjected to Western blot analysis for CD44 and GAPDH. Exosomes isolated from cell culture medium by differential centrifugation were subjected to Western blot analysis for CD44 and an exosome marker CD9. (B) Densitometric analyses for CD44 protein expression in cell lysate (CD44/GAPDH) and exosome (CD44/CD9) were performed in triplicate. Full, non-adjusted images are shown in supplementary material.

**Supplementary Figure 1:**
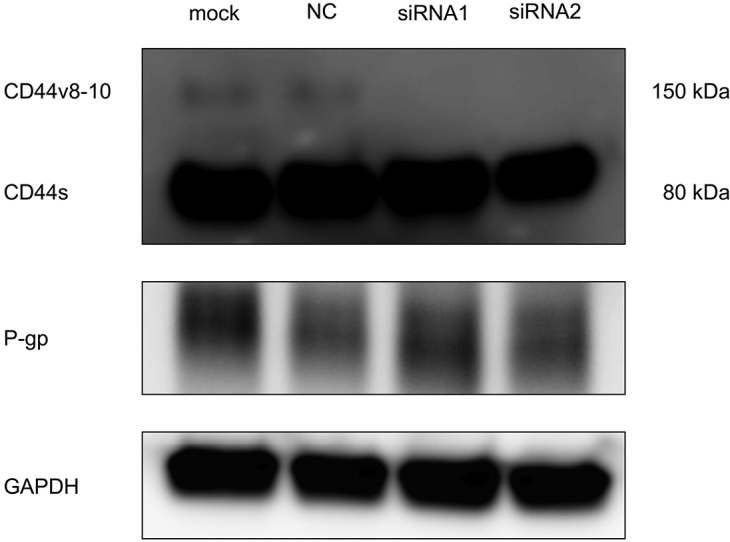
Effects of CD44v 8-10 silencing on P-gp and CD44s expression in PC3-R cells. CD44v8-10 protein expression was specifically silenced by siRNA transfection, whereas expression of P-gp and CD44s was not affected by the knockdown. Full, non-adjusted images are shown in supplementary material.

Western blot analysis using anti-CD44 antibody was performed to determine the CD44 protein levels in cell lysates and exosomes isolated by differential centrifugation from cell culture medium of PC-3R and docetaxel-sensitive PC-3 cells (PC-3 cells) (Figure 1). The level of CD44s was decreased in PC-3R compared to PC-3 cell lysates (0.7-fold, *P* = 0.099), whereas that of the 150 kDa protein was higher in PC-3R cell lysates compared to PC-3 cell lysates (11.8-fold, *P* = 0.001). The protein was identified as a CD44v8-10 variant, because it was silenced by CD44v8-10-specific siRNA transfection (Supplementary Figure 1). The level of CD44v8-10 protein was also higher in PC-3R exosomes than in PC-3 exosomes (75.2-fold, *P* = 0.022). These results indicate that CD44v8-10 protein expression is increased in both cell lysates and exosomes from PC-3R cells compared with those from PC-3 cells.

### 3.2 Increased CD44s and CD44v8-10 mRNA expression in cell lysates and exosomes isolated from PC-3R cells

**Figure 2:**
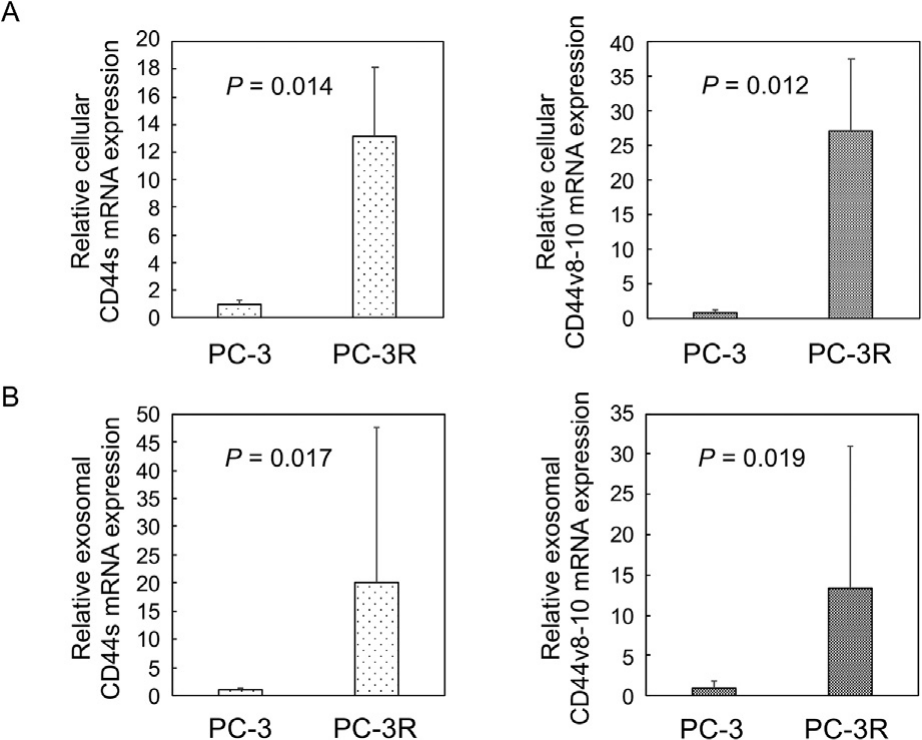
Expression of CD44 mRNA in cell lysates and exosomes isolated from PC-3R and PC-3 cells. Cellular total RNA (A) and exosomal total RNA isolated from cell culture medium using a commercially available kit (B) were subjected to RT-dPCR for CD44s and CD44v8-10 mRNA. Intracellular CD44 mRNA expression was normalized by GAPDH. The CD44s and CD44v8-10 mRNA levels in PC-3R cell lysates and exosomes were higher than those in PC-3 cells (*P* < 0.05, n = 3).

To determine CD44s and CD44v8-10 mRNA levels in cells and exosomes, cellular total RNA was extracted from PC-3R and PC-3 cells and exosomal total RNA was directly extracted from cell culture medium using commercially available kits. After reverse transcription, digital PCR (RT-dPCR) was performed. The levels of CD44s and CD44v8-10 mRNA in PC-3R cell lysates were significantly higher than those in PC-3 cell lysates (13.1- and 27.1-fold, *P* = 0.014 and 0.012, respectively, Student *t*-test, Figure 2A). The CD44s and CD44v8-10 mRNA levels in PC-3R exosomes were also elevated compared with those in PC-3 exosomes (20.1- and 13.3-fold, *P* = 0.017 and 0.019, respectively, Student *t*-test, Figure 2B). These results show that expression of both CD44s and CD44v8-10 mRNA is increased in cell lysates and exosomes from PC-3R cells compared to those from PC-3 cells.

### 3.3 Enhancement of docetaxel sensitivity by CD44v8-10 knockdown in PC-3R cells

**Figure 3:**
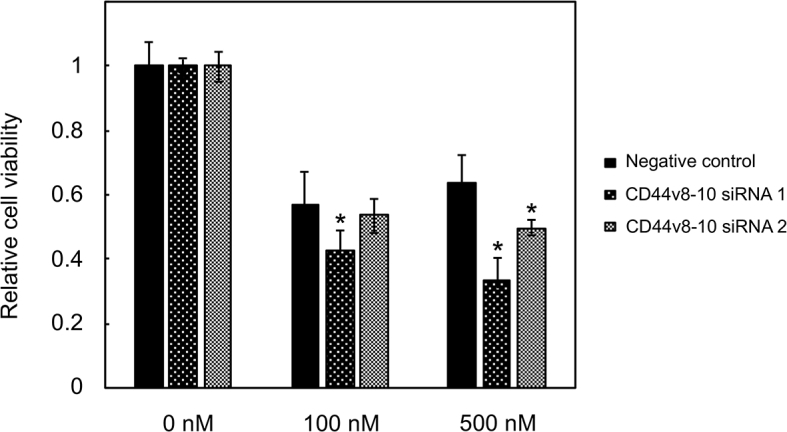
Effect of CD44v8-10 knockdown by siRNA on docetaxel sensitivity in PC-3R cells. CD44v8-10 siRNAs and a negative control siRNA were transfected into PC-3R cells for 6 h. After incubation overnight in complete culture medium, cells (3 × 10^3^) were dispersed, placed in 96-well plates and cultured overnight. After incubation for 72 h in the absence or presence (100 or 500 nM) of docetaxel, WST assay was performed. The relative cell viability after treatment is shown as the percentage of untreated cells. ∗*P* < 0.05 compared to negative control, one-way ANOVA followed by Fisher's LSD test, n = 5.

In order to understand the role of increased expression of CD44v8-10 mRNA and protein in PC-3R cells, the effect of CD44v8-10 knockdown on cell viability was examined after docetaxel treatment for 72 h (Figure 3). Knockdown with one of two CD44v8-10-specific siRNAs enhanced sensitivity to 100 nM docetaxel and both siRNAs significantly enhanced sensitivity to 500 nM docetaxel, respectively.

### 3.4 Increased serum exosomal CD44v8-10 mRNA levels in docetaxel-resistant patients

**Figure 4:**
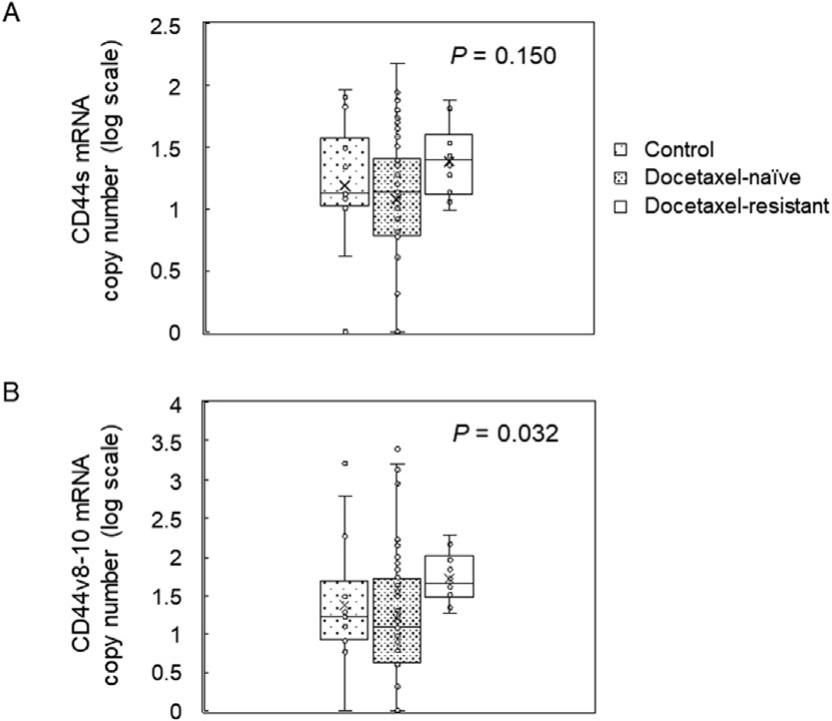
Serum exosomal CD44 mRNA expression in prostate cancer patients. Exosomal total RNA extracted from 500 μL of serum using a commercially available kit was subjected to RT-dPCR for CD44s and CD44v8-10 mRNA. Control males (n = 15), docetaxel-naïve patients (n = 50) and docetaxel-resistant patients (n = 10) were studied. The CD44s mRNA copy number was not significantly different among groups (A) (*P* = 0.150, Kruskal-Wallis test), whereas the CD44v8-10 mRNA copy number was higher in docetaxel-resistant patients compared to control and docetaxel-naïve patients (B) (*P* = 0.032, Kruskal-Wallis test). Data are shown on a log scale because of large variation of mRNA copy numbers.

To determine CD44s and CD44v8-10 mRNA levels in serum exosomes, exosomal total RNA was directly extracted from serum of control males, docetaxel-naïve patients and docetaxel-resistant patients using a commercially available kit. After reverse transcription, dPCR was performed (RT-dPCR). The copy number of CD44s mRNA contained in serum exosomes was not significantly different among docetaxel-resistant patients, docetaxel-naïve patients and control males (median 25, 14 and 13 copies/mL serum, respectively, *P* = 0.150, Kruskal-Wallis test, Table 1 and Figure 4). In contrast, the copy number of serum exosomal CD44v8-10 mRNA was higher in docetaxel-resistant patients compared with docetaxel-naïve and control patients (median 46, 12 and 17 copies/mL serum, respectively, *P* = 0.032, Kruskal-Wallis test, Table 1 and Figure 4).

## 4 Discussion

In the present study, we evaluated CD44 protein and mRNA expression in cell lysates and exosomes isolated from docetaxel-resistant prostate cancer PC-3R cells and parental PC-3 cells. Several CD44 variant isoforms are generated by alternative splicing [10]. Among them, we found that the levels of CD44v8-10 protein and mRNA in cell lysates and exosomes from PC-3R cells were higher than those from parental PC-3 cells (Figures 1 and 2). In contrast, cellular and exosomal CD44s protein levels in PC-3R cells were not increased compared to those in PC-3 cells, despite that their mRNA levels showed marked differences. The mechanism responsible for the discrepancy between CD44s mRNA and protein expression remains to be clarified.

CD44v8-10 protein expression in tumors has been shown to be associated with poor prognosis in various types of cancer [11, 12, 23]. Stefano *et al.* reported that CD44v8-10 is involved in cancer invasion and migration [14], implying its contribution to malignant phenotypes. In terms of drug resistance, CD44 was elevated in ovarian cancer cells and involved in paclitaxel resistance [8]. Hagiwara *et al.* showed that CD44v8-10 expression was upregulated in cisplatin-resistant T24 urothelial cancer cells and its knockdown increased cisplatin sensitivity [13]. These results suggest that CD44 and/or CD44v8-10 may contribute to anti-cancer drug resistance. However, the role of CD44v8-10 in taxane sensitivity in prostate cancer is unknown. In the present study, we demonstrated that knockdown of CD44v8-10 enhanced docetaxel sensitivity in docetaxel-resistant prostate cancer PC-3R cells where its expression was upregulated (Figure 3), suggesting its involvement in docetaxel resistance.

The drug efflux pump P-gp encoded by the *MDR1* gene is considered to be one of the major mechanisms of docetaxel resistance [19]. Bourguignon *et al.* reported that treatment with anti-CD44 antibody decreased MDR1 mRNA and protein levels in breast cancer cells [24]. In contrast, Gao *et al.* reported that knockdown of CD44 was not related to P-gp expression in ovarian cancer cells [8]. We evaluated the effect of CD44v8-10 silencing on P-gp expression and found no correlation between CD44v8-10 and P-gp expression (Supplementary Figure 1). Our results suggest that CD44v8-10 may contribute to drug resistance by other mechanisms than P-gp induction. CD44v8-10 has been reported as a cancer specific stem cell marker in gastric cancer [12, 25] and also shown to be highly expressed in stem-like prostate cancer cells [14]. Furthermore, cancer stem cells play critical roles in chemo-resistance to most anti-cancer agents, the mechanism of which is linked to oxidative stress, drug efflux, DNA repair and epithelial-to-mesenchymal transition [26]. Thus, cancer stem-like feature of the cell lysate and exosome may contribute to docetaxel-resistance in prostate cancer.

Based on our findings that both CD44v8-10 protein and mRNA were upregulated in exosomes from PC-3R cells compared to those from parental PC-3 cells (Figures 1 and 2), we hypothesized that serum exosomal CD44v8-10 could be a diagnostic marker for docetaxel resistance. However, exosomes isolated from serum are highly contaminated with serum proteins [27] potentially including a soluble form of CD44 generated by shedding of the extracellular domain [10]. Since we could successfully measure CD44 mRNA copy numbers in serum exosomes in a quantitative manner by RT-dPCR, we decided to focus on mRNA but not protein of CD44v8-10 in serum exosomes of control and patients. The results showed that serum exosomal CD44v8-10 mRNA was higher in docetaxel-resistant prostate cancer patients than in docetaxel-naïve patients and control males. In contrast, the level of serum exosomal CD44s mRNA did not change significantly among these groups (Table1 and Figure 4). These results suggest that CD44v8-10 mRNA contained in serum exosomes has a potential as a diagnostic marker for docetaxel resistance.

We are aware of the limitations of our study. We directly harvested serum exosomal total RNA using a commercially available kit. Since CD44 is expressed in various tissues, it is likely that we captured exosomes released not only from prostate cancer cells but also from other normal cells, which may in part be responsible for the large variation of exosomal CD44 mRNA copy numbers. The variation may be improved if the mRNA copy number in prostate cancer-specific exosomes could be examined. This study included a small number of docetaxel-resistant patients and did not include docetaxel-sensitive patients during docetaxel administration. A larger analysis including docetaxel-resistant and -sensitive CRPC patients is needed in the future.

## 5 Conclusion

CD44v8-10 may be involved in docetaxel resistance in prostate cancer and serum exosomal CD44v8-10 mRNA could be a diagnostic marker for docetaxel-resistant CRPC.

## Declarations

### Author contribution statement

T. Kato: Conceived and designed the experiments; Performed the experiments; Analyzed and interpreted the data; Contributed reagents, materials, analysis tools or data; Wrote the paper.

K. Mizutani and H. Ehara: Conceived and designed the experiments; Contributed reagents, materials, analysis tools or data.

K. Kawakami, Y. Fujita: Conceived and designed the experiments.

M. Ito: Conceived and designed the experiments; Wrote the paper.

### Funding statement

This research did not receive any specific grant from funding agencies in the public, commercial, or not-for-profit sectors.

### Competing interest statement

The authors declare no conflict of interest.

### Additional information

No additional information is available for this paper.

## Appendix A Supplementary data

Supporting documents Full, non-adjusted images for Figure 1 and Supplementary Figure 1. Supporting documents
