# Supplementary material for: CD44v8-10 mRNA contained in serum exosomes as a diagnostic marker for docetaxel resistance in prostate cancer patients
Source: Heliyon. 2020 Jul 2;6(7):e04138. doi: 10.1016/j.heliyon.2020.e04138 (PMC7334415; doi:10.1016/j.heliyon.2020.e04138)

Supporting documents

Figure 1, CD44s and CD44v8-10 protein expression in cell lysates.

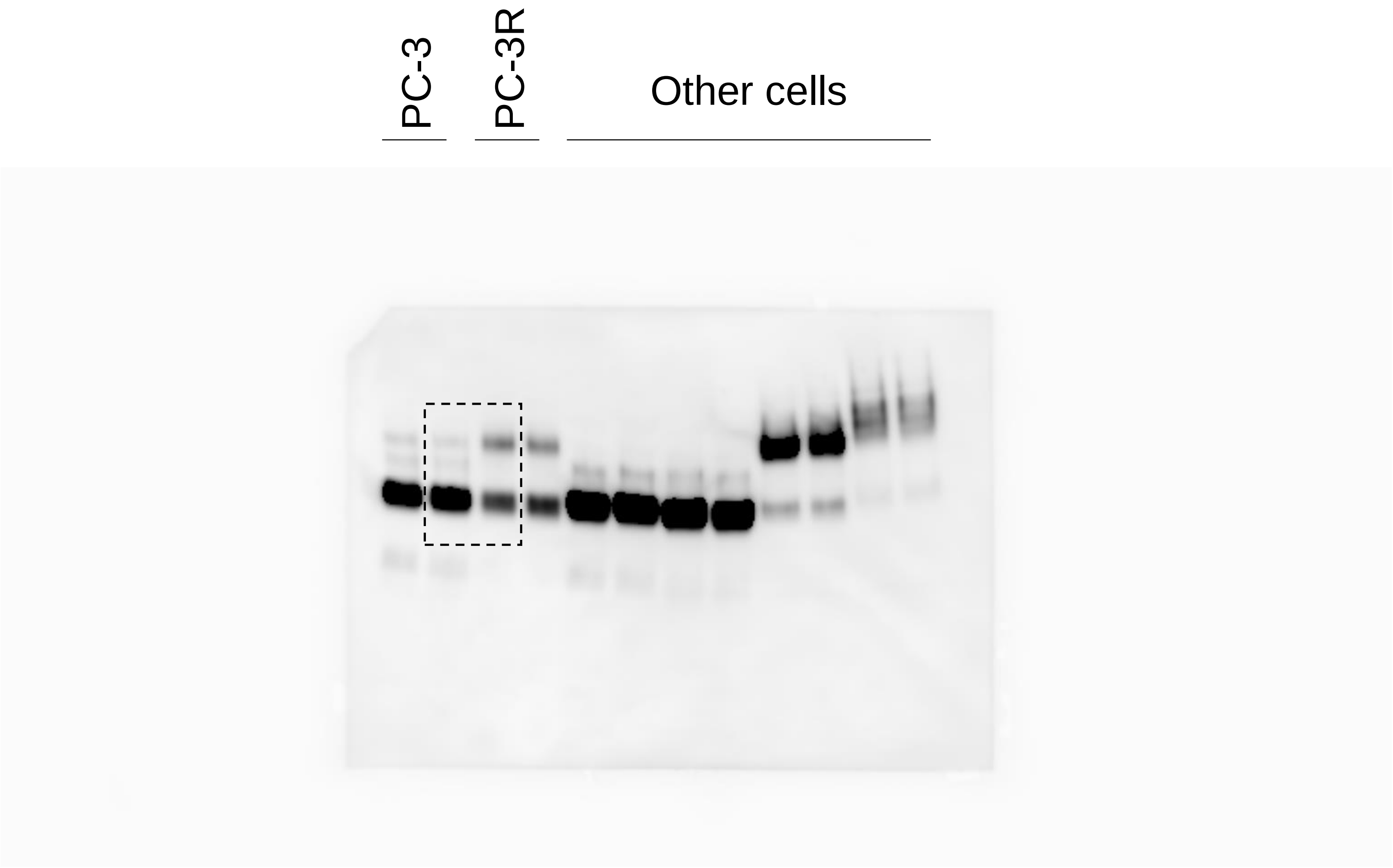

Figure 1, GAPDH protein expression in cell lysates.

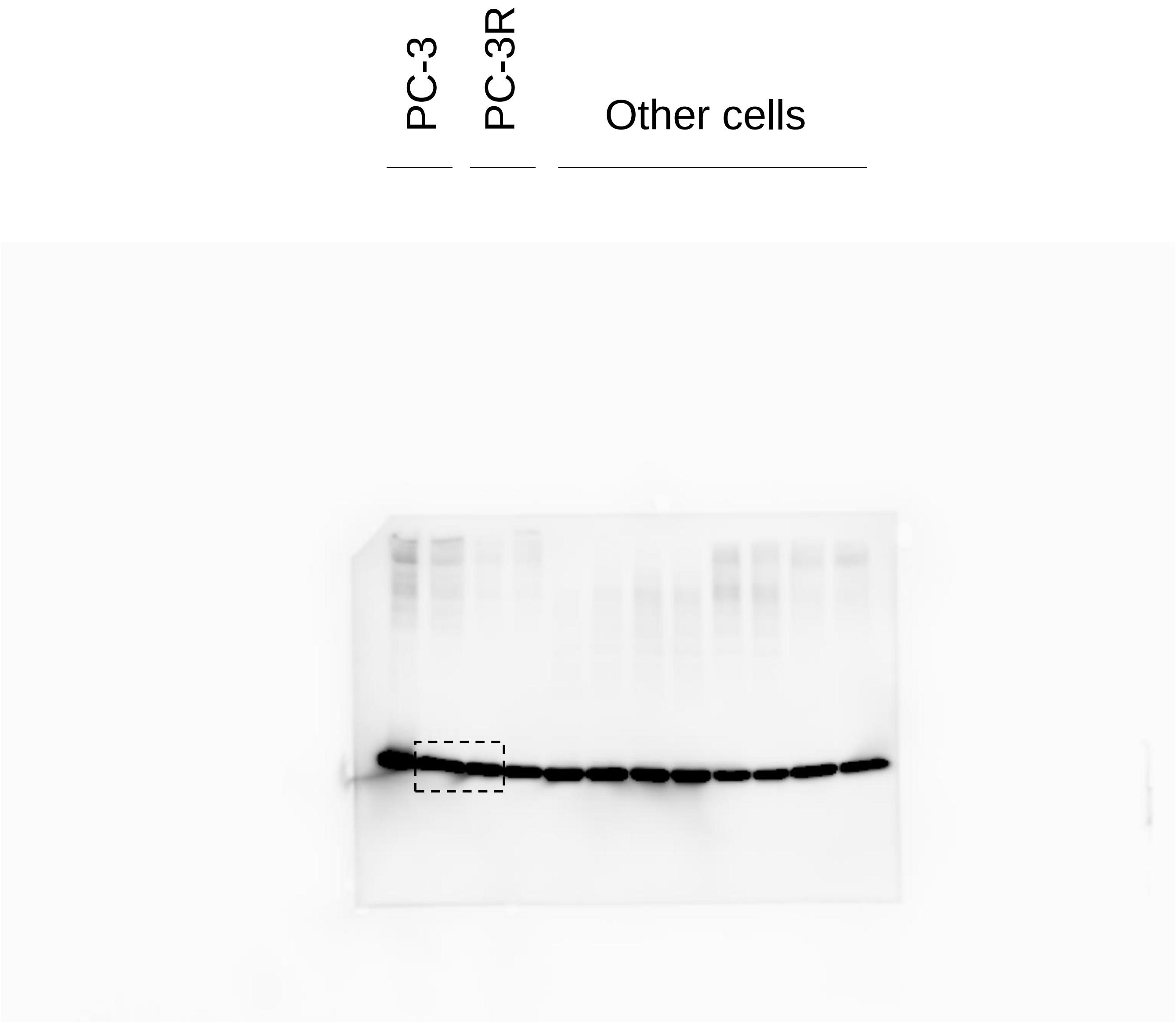

Figure 1, CD44s and CD44v8-10 protein expression in exosomes.

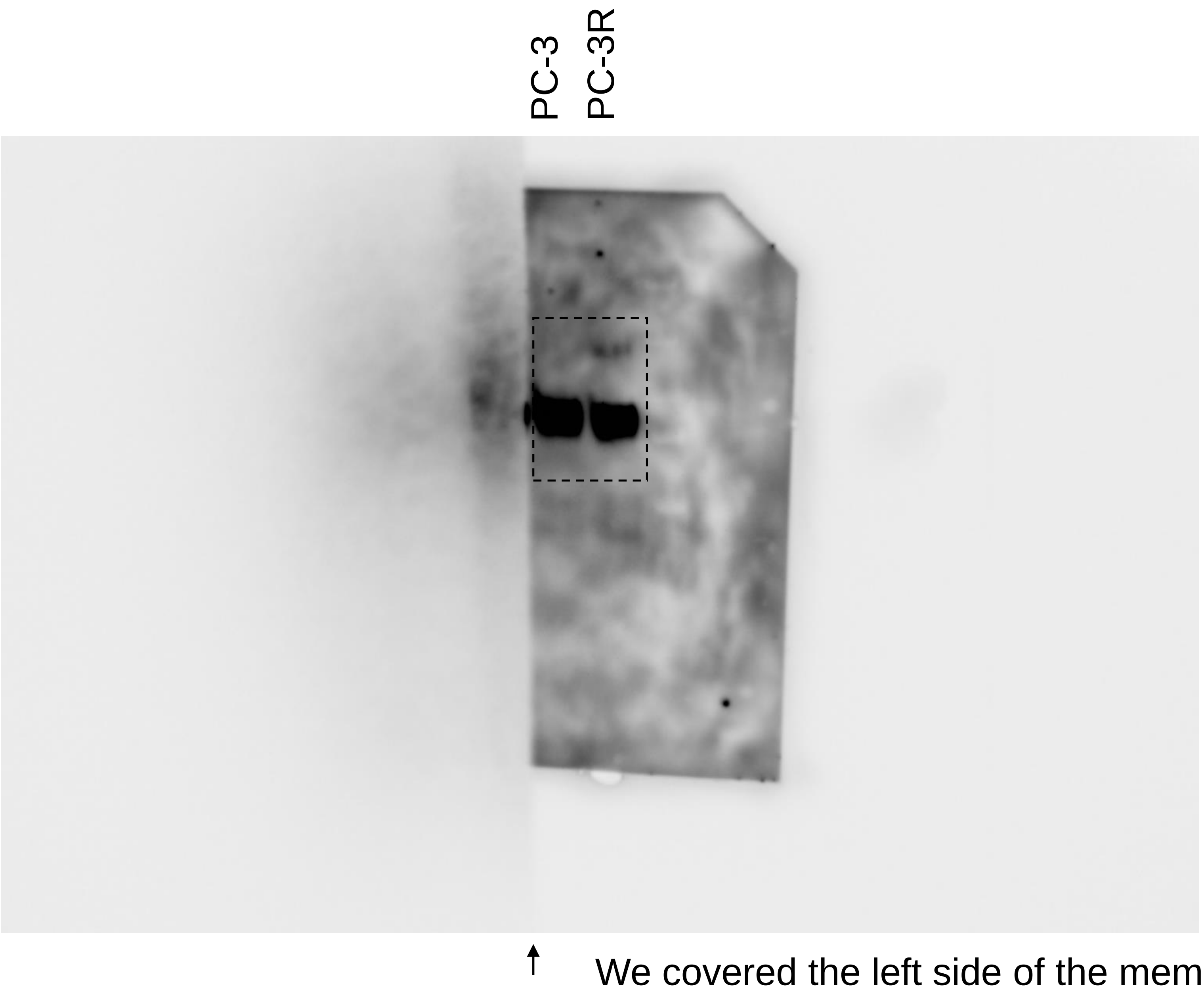

We covered the left side of the membrane because cellular CD44 signal was saturated.

Figure 1, CD44s and CD44v8-10 protein expression on uncovered membrane.

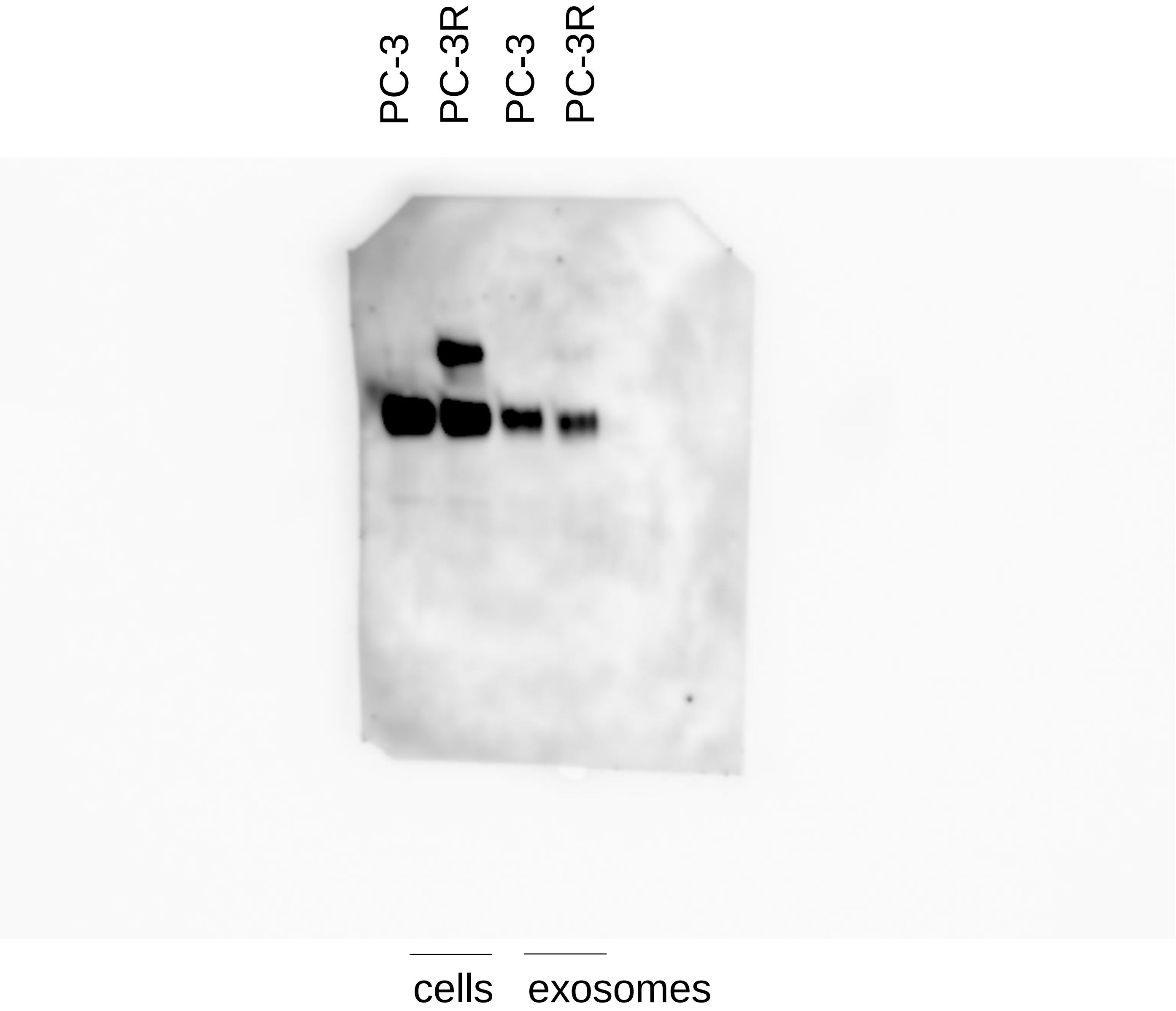

Figure 1, CD9 protein expression in exosomes.

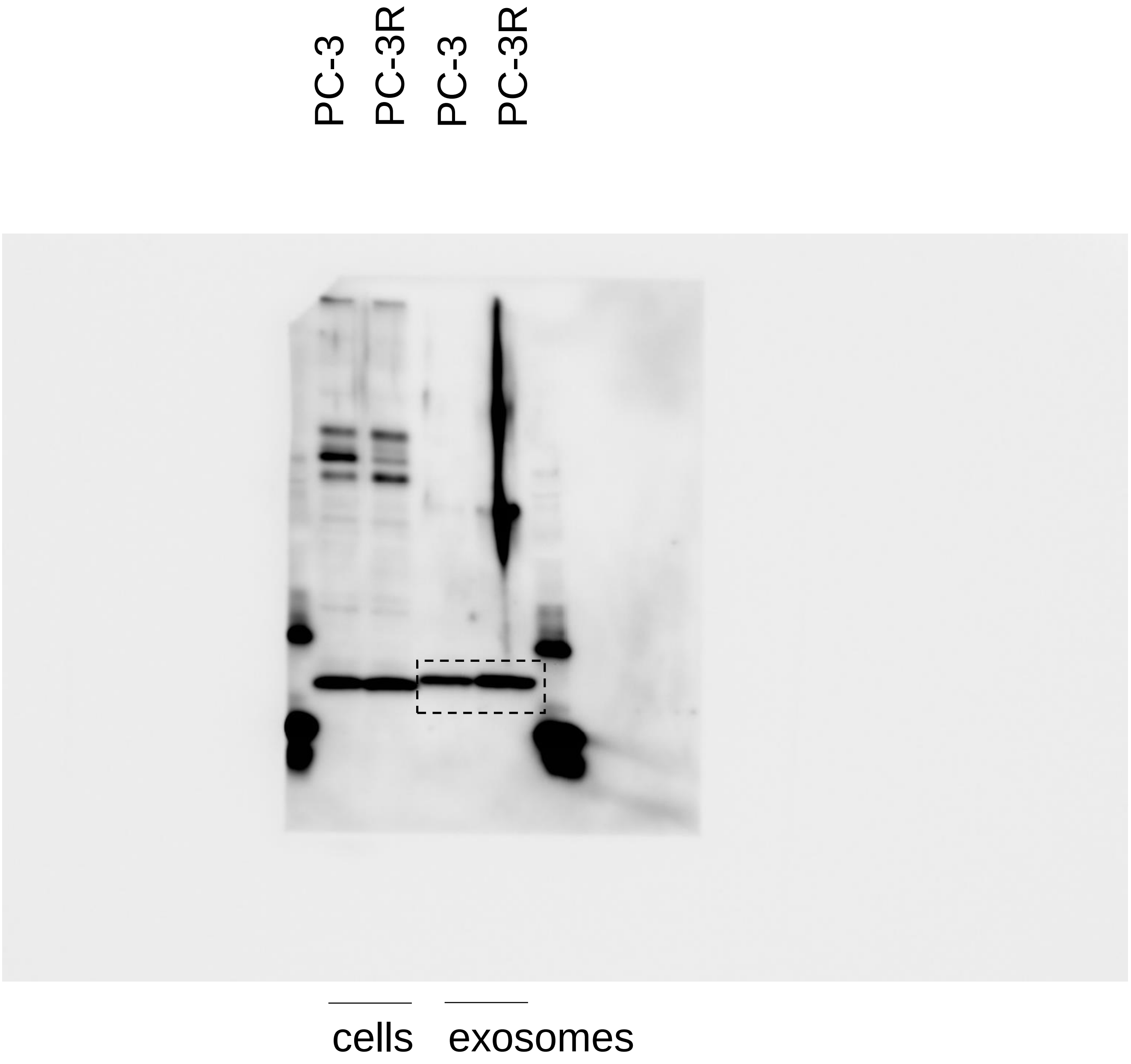

Supplementary Figure 1, CD44s and CD44v8-10 protein expression.

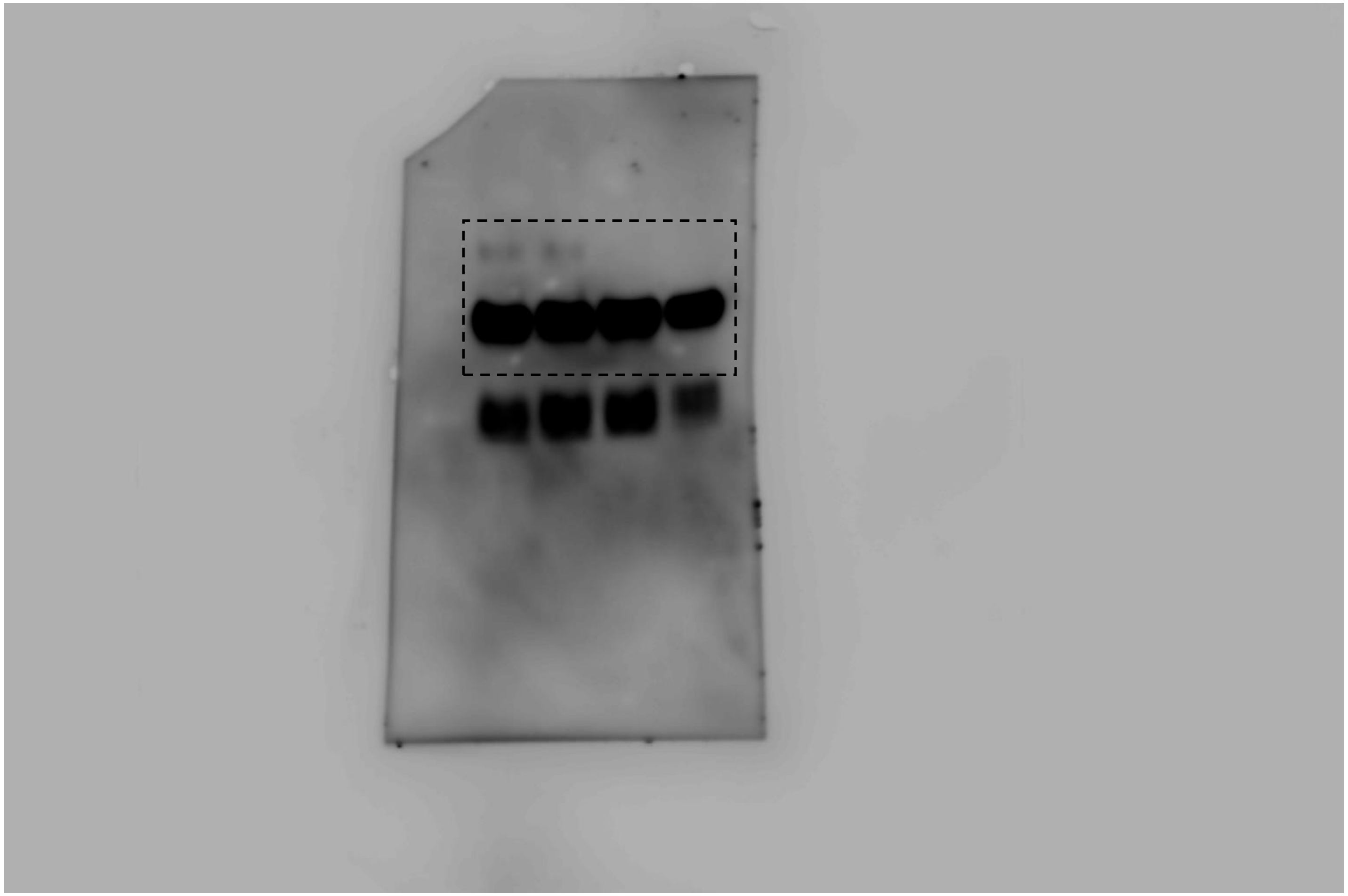

Supplementary Figure 1, P-gp protein expression.

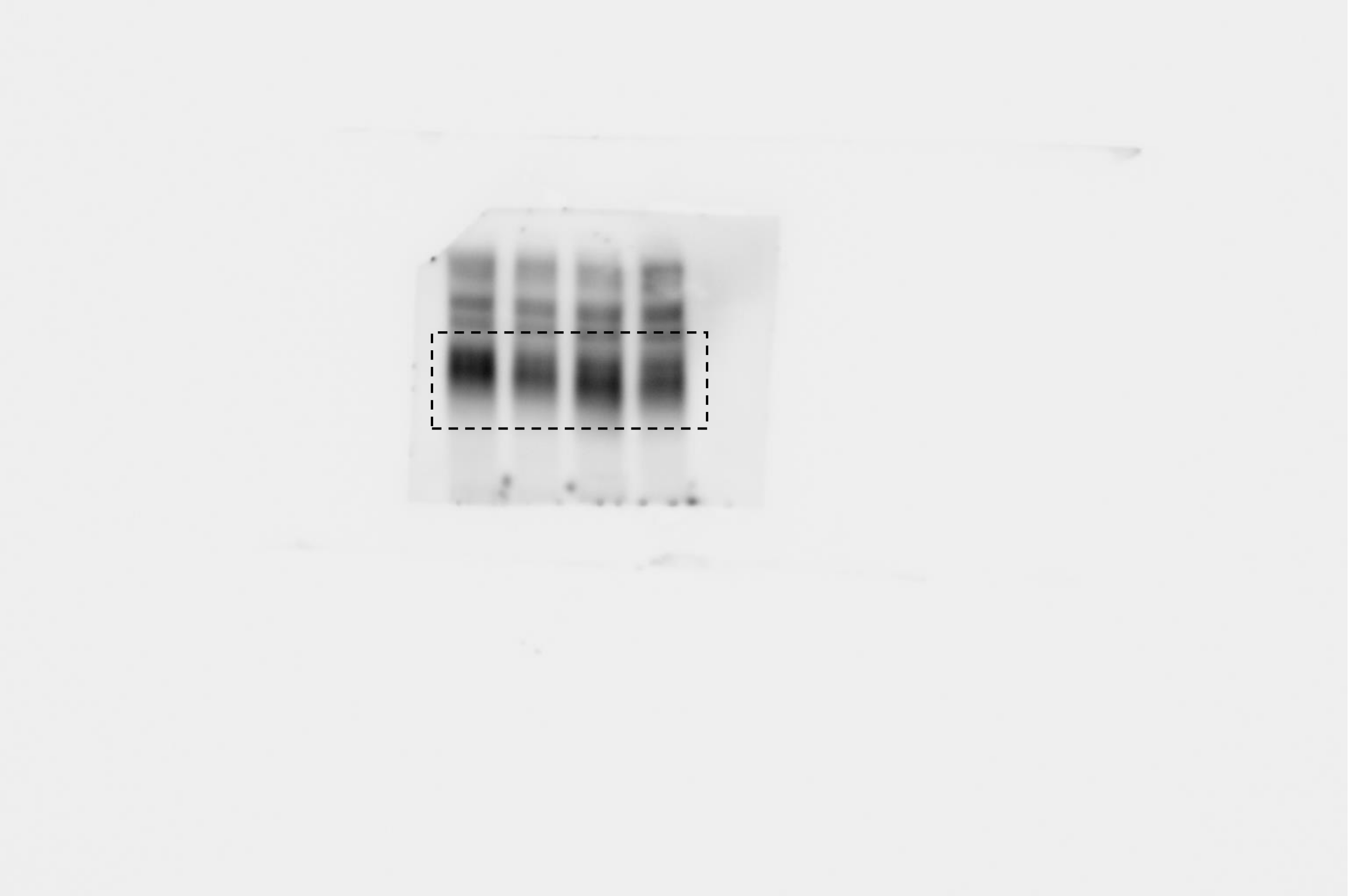

Supplementary Figure 1, GAPDH protein expression.

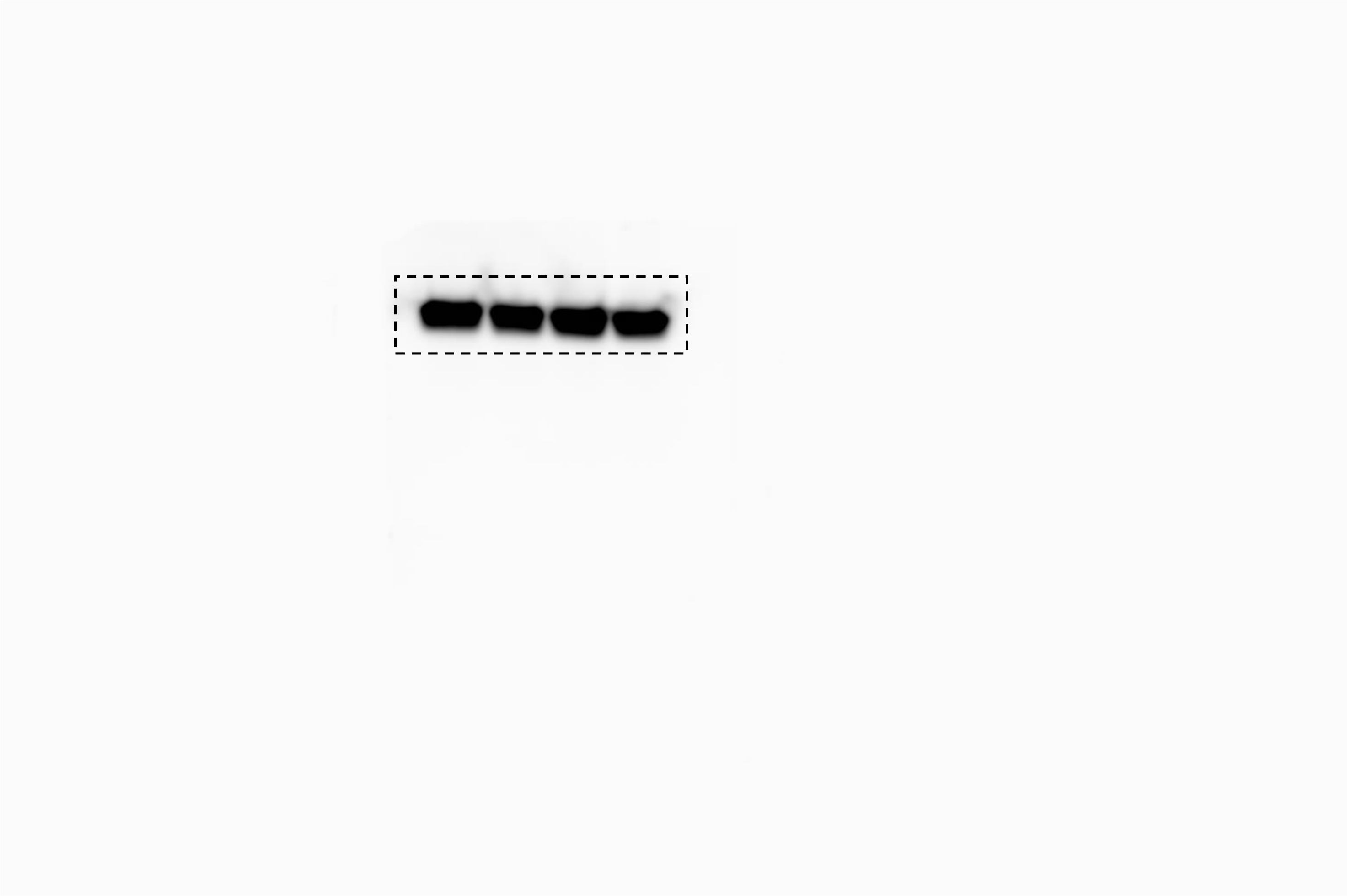

Supplement: Supporting documents — Full, non-adjusted images for Figure 1 and Supplementary Figure 1. [file mmc1.pdf]
